# Effect of composite radiopacity and margin location of the restoration on the diagnosis of secondary caries

**DOI:** 10.1590/0103-6440202405583

**Published:** 2024-03-22

**Authors:** Farzaneh Mosavat, Elham Ahmadi, Faezeh Aghajani, Saeed Ramezani

**Affiliations:** Department of Oral and Maxillofacial Radiology, School of Dentistry, Tehran University of Medical Sciences, Tehran, Iran; 2 Department of Operative Dentistry, Dental research center, Dentistry Research Institute, School of Dentistry, Tehran University of Medical Sciences, Tehran, Iran; 3 Department of Operative Dentistry, School of Dentistry, Tehran University of Medical Sciences, Tehran, Iran; 4 Department of Pediatric Dentistry, School of Dentistry, Tehran University of Medical Sciences, Tehran, Iran

**Keywords:** Radiography, Composite Resins, Dental Caries, Enamel, Dentin

## Abstract

This research aimed to evaluate the effect of the radiopacity of a Bulk-Fill composite (X-TraFil, VOCO, Germany) and a Conventional composite (P60, 3M ESPE, USA) and assessment of the margin location in the enamel and dentin on the diagnosis of secondary caries. 76 intact premolars with MOD preparation were divided into two equal groups and filled with the conventional and bulk-fill composite. Four regions were considered to simulate carious lesions (two regions in enamel and two regions in dentin). In each group, half of the regions in the dentin and half in the enamel were randomly selected for secondary caries simulation and filled with a wax-plaster combination while the remaining regions stayed intact. Bitewing imaging was done using the PSP digital sensor. Five examiners reviewed the images, and lesions were recorded. Caries diagnosis indicators and paired-sample t-test were used for statistical analysis. The reproducibility and accuracy of the examiners’ responses were evaluated using the kappa and agreement coefficient (α=0.05). The sensitivity, specificity, and accuracy of diagnosing secondary carious lesions in enamel were significantly better under conventional than bulk-fill composite. Similarly, the sensitivity and accuracy of diagnosing secondary caries in dentin were significantly higher under conventional composite than bulk-fill composite (p<0.05). No significant differences were found in the agreement and kappa coefficient between conventional and bulk-fill composites in the enamel and dentin (p>0.05). The diagnostic accuracy of carious lesions was higher under conventional composite than bulk-fill composite. However, the location of the secondary was ineffective in caries diagnosis.

## Resumo

Essa pesquisa teve como objetivo avaliar o efeito da radiopacidade de resina composta Bulk-Fill (X-TraFil, VOCO, Alemanha) e de resina composta convencional (P60, 3M ESPE, EUA) e a avaliação da localização da margem no esmalte e na dentina no diagnóstico de cárie secundária. 76 pré-molares intactos com preparo MOD foram divididos em dois grupos iguais e restaurados de acordo com o grupo experimental. Quatro regiões foram consideradas para simular lesões de cárie (duas regiões no esmalte e duas regiões na dentina). Em cada grupo, metade das regiões na dentina e metade no esmalte foram selecionadas aleatoriamente para a simulação de cárie secundária e preenchidas com uma combinação de cera e gesso, enquanto as regiões restantes permaneceram intactas. As imagens de bitewing foram feitas usando o sensor digital PSP. Cinco examinadores analisaram as imagens, e as lesões foram registradas. Os indicadores de diagnóstico de cárie e o teste t de amostra pareada foram usados para análise estatística. A reprodutibilidade e a precisão das respostas dos examinadores foram avaliadas usando o kappa e o coeficiente de concordância (α=0,05). A sensibilidade, a especificidade e a precisão do diagnóstico de lesões cariosas secundárias no esmalte foram significativamente melhores com a resina composta convencional do que com a resina composta bulk-fill. Da mesma forma, a sensibilidade e a precisão do diagnóstico de cáries secundárias na dentina foram significativamente maiores com a resina convencional do que com a resina bulk-fill (p<0,05). Não foram encontradas diferenças significativas na concordância e no coeficiente kappa entre as resinas convencionais e bulk-fill no esmalte e na dentina (p>0,05). A precisão do diagnóstico de lesões cariosas foi maior com a resina composta convencional do que com a resina composta bulk-fill. Entretanto, a localização do secundário foi ineficaz no diagnóstico de cárie.

## Introduction

Dental composites are widely recognized as the leading choice for restorative material on a global scale, primarily employed for their aesthetic. However, despite ongoing advancements, challenges persist, including issues such as overhang, secondary caries, contouring, and adaptation ^1^. Notably, secondary caries stand out as the most frequent long-term complication in dental restorations, often arising from the spaces between the tooth and the restoration. These gaps can be attributed to factors such as polymerization shrinkage, voids within restorations, dissolution of the restorative material at the margins in saliva, or inadequate marginal adaptation ^2^ ^,^ ^3^.

Today, the focus of attention is often directed to the diagnosis and monitoring of secondary caries that develop under restorations, as treating them at early stages is crucial to prevent further damage and preserve the sound structure of the tooth. However, detecting these secondary caries poses a challenge. Dental caries diagnostic methods include visual, tactile, and radiography examinations, with bitewing radiography often employed for detection ^4^ ^,^ ^5^.

Radiopacity is an essential characteristic of restorative materials for diagnosing secondary caries in class I and II restorations, particularly in cervical margins. According to the ISO standard, a material is considered radiopaque if its radiopacity equals or exceeds that of pure aluminum of the same thickness, as its radiopacity is similar to dentin. However, slightly greater radiopacity than that of enamel is required for caries detection in posterior teeth ^6^ ^,^ ^7^. Highly radiopaque materials like dental composites may mask secondary caries due to superimposition, and they can even lead to a false diagnosis of secondary caries due to the Mach band effect. The presence of elements with high atomic numbers, such as barium, strontium, zirconium, and lanthanum in the filler, along with the filler's percentage, increases the radiopacity of composites ^8^ ^,^ ^9^. Enamel caries are expected to be diagnosed with higher accuracy than dentin carious lesions due to the higher opacity of the enamel ^9^.

For the detection of secondary caries, factors beyond the radiopacity of the restorative material and caries location should be considered. The Mach band effect, which produces a radiolucent artifact beneath the restoration in X-ray images due to the difference between the radiopacity of the tooth and restoration, should be acknowledged. This effect can be mistaken for caries ^10^. Furthermore, the radiopacity value may vary depending on the sensor used for imaging ^11^ ^,^ ^12^.

It has been claimed that bulk-fill composites can be light-cured through deeper increments. The X-Tra Fil composite (VOCO, Germany) is a resin-based bulk-fill composite with a filler content of 86 wt%. According to its manufacturing company, it has a radiopacity equal to 330% of aluminum ^13^. Resin-based bulk-fill composites exhibit superior radiopacity compared to conventional composites like P60 (3M ESPE, United States) due to their structure and fillers. However, changes in resin thickness can impact the radiopacity of these composites ^14^.

Studies investigating bulk-fill composites have predominantly focused on evaluating their radiopacity and comparing it with that of conventional composites ^11^ ^,^ ^12^ ^,^ ^13^ ^,^ ^14^. There is a need for more studies that compare these composites in terms of detecting secondary caries. Additionally, studies assessing secondary caries detection under composite restorations have not examined the effect of caries location in the enamel or dentin on secondary caries diagnosis ^1^ ^,^ ^4^ ^,^ ^6^ ^,^ ^15^ ^,^ ^16^. Therefore, the primary objective of this research was to compare the radiopacity of these two types of composites and the location of the restoration cavity margin in relation to secondary caries detection in radiographic images.

## Materials and Methods

Ethical clearance was obtained from the Ethics Committee of Tehran University of Medical Sciences School of Dentistry (IR.TUMS.DENTISTRY.REC.1399.094). Based on the findings of a study by Crus et al. ^1^ and utilizing the "test for paired sensitivities" of PASS software version 15, the minimum sample size required for the study was determined to be 76 samples, assuming P=0.5, sens1=0.733, sens2=0.983, ꞵ=0.2, α=0.05, and a proportion discordant=0.3.

Seventy-six intact human premolars without any cracks, decalcification, caries, or fractures, extracted for orthodontic or periodontal reasons, were utilized in this study. All tooth surfaces underwent prophylaxis with a rubber cup and pumice. Following prophylaxis, the teeth were disinfected by immersing them in Chloramine-T for one week and subsequently stored in distilled water at 4˚C until further use. The materials employed in this study are detailed in Box 1.

**Box 1:**
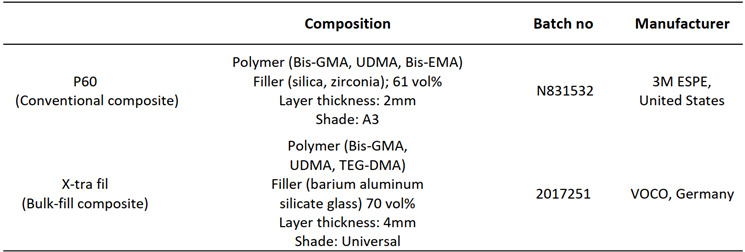
Materials used in this study

The teeth were first mounted on a wax block (Figure 2A). Then, MOD preparation was performed, with proximal boxes measuring 2 mm in width and 3 mm in length and depth, and occlusal boxes measuring 2 mm in width and 1.5 mm in depth by a dentist using a high-speed turbine and copious air-water spray with a diamond bur (V835/010 - Flat End Cylinder) and checked with a probe ^1^ ^,^ ^6^.

**Figure 1:**
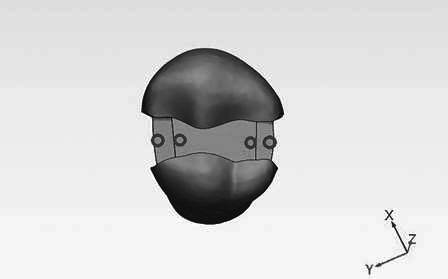
Candidate regions for caries production. Points A are considered to simulate enamel caries in the gingival floor of the proximal boxes. Points B are considered to simulate dentin caries in the pulpal floor of the occlusal boxes.

A random number table was used to assign the teeth equally to the X-Tra Fil composite (VOCO-Germany) (B) and P60 composite (3M, ESPE, United States) (C) composite groups. On each tooth, four regions were considered for the simulation of carious lesions, including A) two regions in the mesiogingival and distogingival line angles in the middle of the floor of the distal and mesial aspects of proximal gingival boxes in the enamel and B) two regions in the pulpal floor of the occlusal box on the mesial and distal aspects of the tooth in the dentin. In each group, half of the regions in the dentin and half in the enamel were randomly selected for secondary caries simulation using a random numbers table. In contrast, the other half remained intact (control group). The location of the carious lesions was random; in other words, both proximal boxes of a tooth or both mesial and distal aspects of the occlusal box of a tooth were intact or carious, or one aspect was carious while the other aspect was intact (Figure 1).

**Figure 2:**
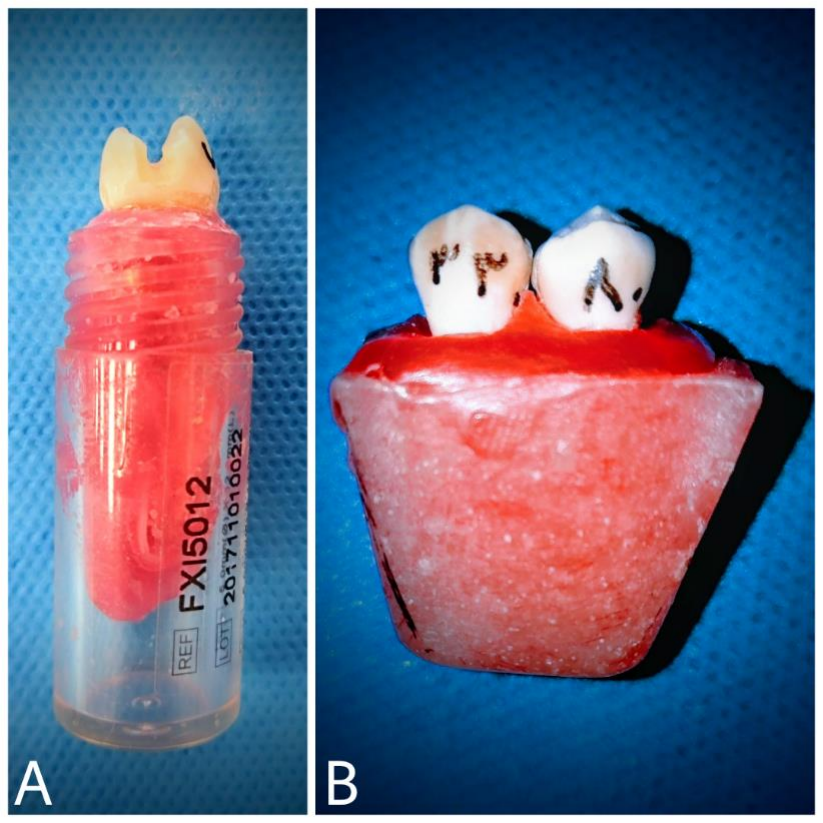
2A) The wax block is used for the preparation and restoration of teeth. 2B) The acrylic blocks for radiographic imaging.

On the regions selected for secondary caries production, a cavity measuring 1 mm in width and 0.5 mm in depth was prepared using a #1 round carbide bur. The cavities were filled with a wax-plaster combination at a ratio of 3 to 1 to reduce the contrast of the lesion and make it more realistic ^4^. A Mylar matrix strip with a matrix holder was placed. All restoration surfaces were etched with 35% phosphoric acid (Vococid, Voco, Germany) for 15 seconds and then washed for 20 seconds, and excess moisture was removed with absorbent paper. Next, Solobond M (VOCO, Germany) was applied to all cavities according to the manufacturer's instructions and light-cured at an intensity of 1000 MW/cm² for 20 seconds using the DTE LUX-E curing light (Woodpecker, PRC). According to the manufacturer's instructions, half of the teeth were filled with 4-mm thick layers of X-Tra Fil composite (VOCO-Germany) and light-cured for 10 seconds. The other half was filled with 2-mm thick layers of P60 composite (3M, ESPE, United States) and light-cured for 20 seconds.

For X-ray imaging, the teeth were randomly mounted on cold-cured acrylic blocks (ACROPARS, Iran) so that two premolar teeth were mounted next to each other and were in contact. The contact quality was checked with dental floss. The teeth were mounted in such a way that 2 mm of the root surface was exposed for soft tissue simulation, and two layers of red wax were applied around the teeth (Figure 2B).

Imaging was done using the Owandy-RX radiology system (Owandy, France) (kvp=60, distance= 40 mm, time= 0.25 seconds). Bitewing imaging was taken using XCP so that the beam was perpendicular to the longitudinal axis and in contact with the teeth (0˚ horizontal angle). Imaging was done using the PSP digital sensor. The images were obtained using the Digora Optime dental phosphor plate processor (Soredex, Finland) and coded according to the number of each tooth ^1^ ^,^ ^6^.

**Figure 3:**
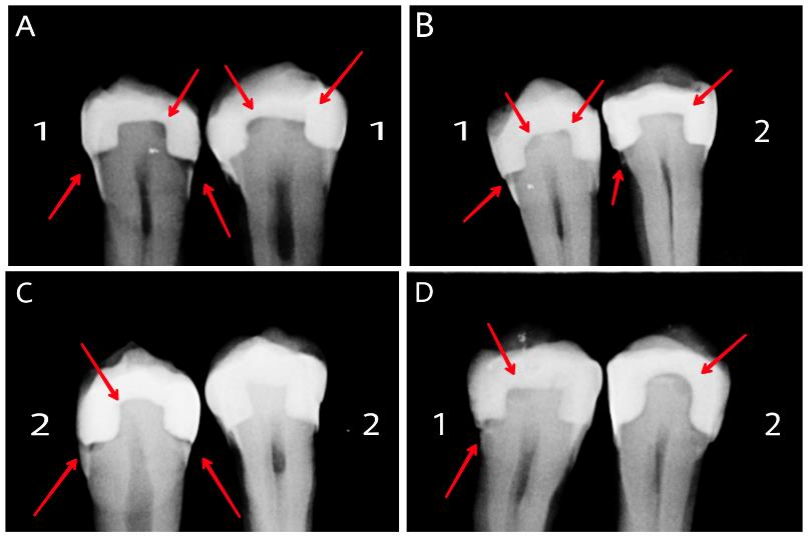
3A-3D depicts radiographic images, with No. 1 corresponding to P60 fillings and No. 2 representing X-Tra Fil fillings. Arrows indicate simulated caries in the gingival floor of the proximal boxes and the pulpal floor of the occlusal boxes.

Five examiners, including one oral and maxillofacial radiologist and four operative dentistry specialists unaware of the samples, evaluated the images in a low-light room with similar laptops (Modern 15 MU, MSI, Taiwan). A dual scale (1: positive for caries, 2: negative for caries) was used to assess the images. The results were entered into a checklist that contained the number of teeth and candidate caries regions from the mesial aspect to the distal aspect in the form of empty boxes. After one week, two examiners were asked to evaluate the images to measure reliability. The examiners had no time limits and could adjust the brightness, contrast, and zoom in and out ^6^ (Figure 3).

The sensitivity, specificity, and accuracy of secondary caries diagnosis were measured. Statistical comparisons based on the composite type and caries location were conducted using the paired samples t-test. The reproducibility and accuracy of the examiners' responses were evaluated using the Kappa test and agreement coefficient. Statistical analysis was performed using SPSS 23, with p-values less than 0.05 considered significant.

## Results

Data analysis was conducted using SPSS 23 software. The mean and standard deviation values of diagnostic tests for the assessment of images by examiners regarding secondary caries diagnosis were calculated. Additionally, the agreement and kappa coefficients of the examiners were measured based on the re-examination results (Table 1).

Comparison of the sensitivity, specificity, and accuracy of secondary caries diagnosis, considering the composite type and caries location, revealed that the sensitivity, specificity, and accuracy of diagnosing secondary caries in the enamel were significantly better beneath the conventional composite compared to the bulk-fill composite (p < 0.05). Similarly, the sensitivity and accuracy of diagnosing secondary caries in the dentin were significantly better beneath the conventional composite than the bulk-fill composite. Nonetheless, no difference was found in the specificity of secondary caries diagnosis between these two composites (p > 0.05). Additionally, no significant difference was found in the sensitivity and accuracy of secondary caries diagnosis in the enamel versus dentin beneath conventional composite (p > 0.05). However, the difference in their specificity was significant (p < 0.05). Similarly, no significant difference was found in caries diagnostic tests in the enamel versus dentin beneath the bulk-fill composite (Tables 2 and 3).

**Table 1:** The Mean and standard deviation values of the diagnostic testing for observers' performance in detecting secondary caries-like lesions.

Samples	Conventional		Bulk fill	
	Enamel	Dentin	Enamel	Dentin
Accuracy	0.93±0.03	0.87±0.09	0.86±0.03	0.83±0.13
Sensitivity	0.87±0.07	0.81±0.13	0.77±0.11	0.73±0.15
Specificity	0.99±0.01	0.93±0.06	0.92±0.03	0.93±0.12
Agreement coefficient	0.95±0.01	0.94±0.01	0.96±0.01	0.96±0.02
Kappa coefficient	0.90±0.02	0.87±0.03	0.93±0.02	0.93±0.05

**Table 2:** Comparison based on composite type (P60 compared to X-tra fil) by paired sample t-test.

		Mean	Standard deviation	P-value
Enamel (P60 - X-tra fil)	Sensitivity	0.10	0.13	0.042*
	Specificity	0.07	0.03	0.000*
	Accuracy	0.07	0.04	0.001*
Dentin (P60 - X-tra fil)	Sensitivity	0.08	0.06	0.003*
	Specificity	0.00	0.07	0.963
	Accuracy	0.04	0.05	0.029*

* significance values.

Positive values indicate the superiority of the P60 composite and negative values indicate the superiority of the x-trafil composite.

**Table 3:** Comparison based on secondary caries location (enamel compared to dentin) by paired sample t-test.

		Mean	Standard deviation	P-value
P60 (Enamel - Dentin)	Sensitivity	0.05	0.14	0.235
	Specificity	0.06	0.06	0.014*
	Accuracy	0.06	0.10	0.089
X-tra fil (Enamel - Dentin)	Sensitivity	0.03	0.22	0.594
	Specificity	- 0.01	0.14	0.841
	Accuracy	0.03	0.15	0.508

* significance values.

Positive values indicate the superiority of Enamel and negative values indicate the superiority of dentin.

## Discussion

Secondary caries are one of the most common reasons for the failure of composite restorations. Radiography is often employed to diagnose these secondary caries ^5^. Sufficient radiopacity of a restorative material, resulting from a contrast difference, makes it possible to differentiate between enamel and dentin. Moreover, it facilitates the diagnosis of secondary caries, the presence of voids in the interface, excessive restorative material, and inadequate adaptation of the restorative material, especially in the posterior teeth ^6^.

The present study demonstrated that bulk-fill and conventional composites did not offer similar diagnostic accuracy for secondary caries in bitewing images due to differences in their radiopacities, with conventional composites exhibiting better performance. Additionally, the diagnostic accuracy of secondary caries beneath bulk-fill and conventional composites was not influenced by the margin location in the enamel and dentin in bitewing images.

Similar to a study by Anbiaee et al. ^4^, a wax-plaster combination at a ratio of 3 to 1 was used for the simulation of carious lesions. However, artificially simulating caries may not precisely recreate the conditions of real caries, representing one of the limitations of studies addressing secondary caries. In contrast to the aforementioned study, premolar teeth with similar dimensions were chosen for sample homogenization instead of molar teeth, which have rather heterogeneous shapes and dimensions. The use of premolar teeth aimed to minimize variations that could impact caries diagnosis. Additionally, for imaging purposes, the teeth were mounted with their proximal surfaces in contact, simulating their contact and soft tissue to replicate intraoral conditions ^4^ ^,^ ^6^.

The radiopacity of dental composites is directly correlated with their composition, encompassing the type and quantity of fillers, as well as the density and thickness of the material ^8^. Numerous studies have demonstrated that the bulk-fill composites commercially available adhere to ISO standards concerning radiopacity. Nevertheless, the radiopacity of bulk-fill composites notably increases with a rise in thickness. However, in the current study, composite thickness played no role in the visual examination due to the similar dimensions of the boxes. The primary determinant of radiopacity in composites is the fillers. The types and percentages of these fillers directly influence the radiopacity of a composite ^11^ ^,^ ^14^ ^,^ ^17^.

For a long time, it was recommended to insert composites using 2-mm layers to reduce polymerization stress and improve mechanical properties ^18^ ^,^ ^19^ ^,^ ^20^. However, due to the time-consuming nature of this method, bulk-fill composites, designed to be cured in thicker layers with reduced polymerization stress, were introduced to the market. These composites gained rapid popularity among dentists for their ability to reduce the time required for restoring large cavities, simplifying the process ^21^. In the current study, a bulk-fill composite (X-Tra Fil, VOCO, Germany) with a 70 vol% filler content (barium aluminum silicate glass) and a conventional composite with a 61 vol% filler content (zirconium oxide and silica) were utilized ^11^ ^,^ ^13^. As anticipated, X-Tra Fil exhibited higher radiopacity, as evident in X-ray images.

In this study, the P60 composite, with a radiopacity value slightly higher than that of enamel, demonstrated significantly better sensitivity and specificity in diagnosing secondary caries compared to the X-Tra Fil composite, which had higher radiopacity. This finding aligns with the results of a study by Cruz AD et al. ^1^, which investigated four composites, including Charisma (Heraeus Kulzer GmbH, Germany), Filtek Z250 (3M ESPE, USA), Prisma AP. H (Dentsply International Inc, Brazil), and Glacier (SDI Limited Bayswater, Australia). The study found that composites with higher radiopacity values had lower diagnostic accuracies, while composites with lower radiopacity values (though still more opaque than dental tissue) offered reasonable diagnostic accuracy. In this study, carious lesions were simulated on the floor of the distal and mesial proximal gingival boxes (dentin margin). High radiopacity may interfere with an accurate diagnosis of secondary caries, and the optimal radiopacity value for diagnosing caries is close to that of intact dentin. However, Pedrosa et al. ^6^ evaluated one conventional and four flowable composites, concluding that radiopacity values at least close to those of enamel favored the diagnosis, while lower radiopacity values confused the examiner. In the present study, both composites had radiopacity values higher than that of the enamel. Therefore, selecting composites with radiopacity values lower than that of the enamel for the initial restorative level may lead to inaccurate interpretations of carious lesions in the enamel ^23^ ^,^ ^24^ ^,^ ^25^.

Moreira et al. ^26^ investigated three composites, Charisma (Heraeus-Kulzer, Germany), Z250-Filtek (3M ESPE, USA), and Spectrum-TPH (Dentsply, USA). Spectrum-TPH (Dentsply, USA) exhibited the highest radiopacity and the lowest diagnostic accuracy, which aligns with the findings of the present study. Very high radiopacity values may interfere with a correct diagnosis and induce the Mach band effect ^10^. Therefore, manufacturers should consider the appropriate radiopacity of dental composites, and clinicians should carefully select the proper composite for early and accurate diagnosis of secondary caries.

The type of sensor used for imaging is a critical factor in evaluating the radiopacity of the material. Although Cruz AD et al. ^1^ reported similar results, they utilized analog imaging in their study. However, previous studies investigating secondary caries diagnosis showed no significant differences in the accuracy of digital and conventional imaging systems. It should be noted that CCD and PSP systems were used in these studies ^4^ ^,^ ^6^. Yasa et al. ^11^ found that the CMOS sensor was more sensitive to details than other imaging methods, possibly due to the higher spatial resolution of this sensor.

Another crucial factor to consider when evaluating the radiopacity of the material and diagnosing secondary caries is the experience of observers in detecting secondary caries. A previous study highlighted the absence of guidelines for accurately diagnosing secondary caries in daily clinical practices ^27^. Despite the limited accuracy of bitewing images for secondary caries diagnosis, it is recommended as a tool for periodic assessment of the presence of secondary carious lesions (in conjunction with physical examination) to aid in making a more accurate diagnosis ^28^. Consistent with earlier studies, the findings of the current study, utilizing a simulated model of secondary caries, underscored the significance of image perception over the observers' experience ^1^ ^,^ ^6^ ^,^ ^27^. Furthermore, the reliability pattern of the examiners' responses indicated no notable difference between conventional and bulk-fill composites in the enamel and dentin.

Based on the findings of the present study, the accuracy, sensitivity, and specificity of diagnosing secondary caries were slightly higher in the enamel compared to the dentin for both composites. However, the difference was not statistically significant, except for the specificity of diagnosing secondary caries in the enamel under conventional composite, which was significantly better than for secondary carious lesions in the dentin. Previous studies investigating secondary caries diagnosis under various composite restorations did not assess the impact of caries location in the enamel and dentin on the diagnosis ^1^ ^,^ ^4^ ^,^ ^6^ ^,^ ^15^ ^,^ ^16^. Given the higher radiopacity of enamel, it was expected that diagnosing secondary caries would be easier in enamel lesions. The study's results supported this expectation, although the difference was not statistically significant.

It is important to highlight that the results of laboratory studies, such as the present one, may not be directly applicable to the diagnosis of secondary carious lesions in the oral cavity due to the more complex challenges present in clinical settings. In the oral environment, diagnosing caries developed under composite restorations is influenced by various factors, including radiography-related aspects such as the size of the lesion, type of sensor or film, radiography unit settings, as well as personal factors like the experience and skill of the clinician and the availability of suitable equipment such as a quality monitor. These complex interactions should be considered in future studies designed to investigate the impact of secondary caries' location in the enamel and dentin in different restorations. The design of such studies should carefully account for the combined effects of confounding variables on the diagnosis of secondary caries, as mentioned earlier.

## Conclusion

The type of composite used can indeed influence the diagnosis of secondary caries beneath restorations, emphasizing the importance of selecting restoration materials with appropriate radiopacity for accurate diagnosis. However, in this study, the location of caries in the enamel versus dentin did not show a significant effect on the diagnosis.

Clinical relevance**:** The choice of composite material has a notable impact on the diagnosis of secondary caries, underscoring the importance of selecting appropriate restorative materials. However, in this study, the location of caries in the enamel versus dentin did not demonstrate a significant correlation with the diagnosis.
